# Chromatic Coloration Mechanism of Nigerian Blue-Green Beryl: Multi-Ion Synergistic Effects

**DOI:** 10.3390/ma19214470

**Published:** 2026-10-23

**Authors:** Jingwen Pang, Geng Li, Fabian Dietmar Schmitz

**Affiliations:** 1 School of Gemology, China University of Geosciences (Beijing), Beijing 100083, China; 2109250033@email.cugb.edu.cn; 2 Rhein Main Gem Consulting, Forsterstraße 4, 55118 Mainz, Germany; fabian.d.schmitz@googlemail.com

**Keywords:** beryl, Nigeria, coloration mechanism, multi-ion synergy, chromaticity

## Abstract

The blue-green “electro-optical” green beryl from Nigeria has gained increasing commercial attention, yet its specific coloration mechanism remains unclear. This study investigates ten samples using XRF, UV–Vis–NIR, IR, and Raman spectroscopy to characterize their chemical compositions, spectral features, and coloration origins. The chromophoric elements are predominantly Fe, Cr, and V, with Fe significantly higher than Cr and V. UV–Vis–NIR spectra reveal that the blue-green hue results from synergistic absorptions of V^3+^/Cr^3+^ in the yellow-green region, Fe^2+^–Fe^3+^ intervalence charge transfer (IVCT) in the red region. Chromaticity analysis shows centroid color differences ΔE*ab of 7.31 between “V-bearing“ and “V not detected” groups, and 20.39 between “high-TFeO/(Cr_2_O_3_ + V_2_O_5_)” and “low-TFeO/(Cr_2_O_3_ + V_2_O_5_)” groups, both far exceeding the visual perceptibility threshold. This study provides quantitative spectroscopic and chromaticity constraints for understanding the multi-ion synergistic coloration of Nigerian blue-green beryl.

## 1. Introduction

Beryl (Be_3_Al_2_Si_6_O_18_) is a cyclosilicate characterized by a channel-rich structure, within which traces of transition metal ions give rise to a wide variety of colors [1,2]. Iron is the most common and complex chromophore, but its site occupancy and valence states in beryl remain debated. Chromium and vanadium substitute for octahedral Al and are responsible for green to yellowish-green colors characteristic of emerald [3,4]. The Nigerian blue-green beryl samples investigated in this study originate from the Jos Plateau–Kaduna region in central Nigeria. In this region, emerald and green beryl mineralization is associated with the Mesozoic Jos ring complex, where alkaline granites and associated pegmatites intruded the Nigerian Basement Complex [5,6]. Emerald formation is related to early metasomatic albitization of the alkaline granite and occurs in small pegmatitic pockets and miarolitic cavities, generally within 20 m of the contact between the granite and the overlying basement rocks. The chromophore Cr was likely derived from the basement schists or younger volcanic rocks, whereas Be was supplied by the alkaline granitic–pegmatitic system [6]. Fluid inclusion studies indicate that emerald crystallized at approximately 400–450 °C and 0.2–0.3 kbar during the early and intermediate growth stages [7,8].

In recent years, a blue-green “electro-optical” beryl from central Nigeria and the Jos Plateau pegmatites has entered the international market. The blue-green “electro-optical” beryl refers to a variety of beryl that exhibits a blue-green color with high brightness, high saturation, and a slight neon-like quality, with a hue between the blue of aquamarine and the green of beryl. The term “electro-optical” here is a visual/commercial descriptor, indicating a bright, vivid, and striking color. Commercial laboratories often classify it as emerald, but its mineralogical nomenclature is unsettled; this study tentatively refers to it as blue-green beryl. Hong et al. [9] reported that three Nigerian samples are dominated by Fe, followed by V with V > Cr, and proposed a color model combining V^3+^-induced yellowish-green with Fe^2+^–Fe^3+^ IVCT-induced light blue. However, V^3+^ and Cr^3+^ differ systematically in crystal-field splitting and band half-widths, so a simple superposition model cannot fully explain the observed hue variations. A comprehensive spectroscopic, chemical, and chromaticity study is therefore needed to clarify the coloration mechanism of this beryl type.

## 2. Materials and Methods

Ten Nigerian beryl samples (A-B-1, A-B-2, A-B-4, A-B-6, A-B-8, C-1, C-2, C-3, C-4, C-5) were selected. All are blue-green, transparent to translucent, comprising four uncut crystals and six faceted stones, weighing 0.259 to 2.773 ct. Preliminary tests confirm their gemological parameters are consistent with standard beryl values. Characteristic inclusions were observed in samples such as C-5.

Standard gemological methods were employed to comprehensively examine the optical properties of the samples, including microscopic features, refractive index (RI), birefringence (DR), dichroism, ultraviolet fluorescence. Microscopic features were observed using a gemological microscope. Dichroism was measured with a calcite dichroscope. Ultraviolet fluorescence observations were conducted using UV lamps operating at 365 nm (long-wave) and 254 nm (short-wave). These instruments were manufactured by Baoguang Technology (Nanjing, China). Refractive index and birefringence were measured using a Gem-A refractometer produced by the same company.

The color measurement instrument used was the MDIS-f8 multi-function double integrating sphere spectrometer manufactured by LiuLab (South El Monte, CA, USA). Its specially designed double integrating sphere optical system provides optimal illumination and geometric conditions for spectral reflectance and color measurement of multi-faceted colored gemstones and diamonds under controlled conditions. To ensure accuracy in spectral and color measurements, the spectrometer employs an innovative three-step calibration method: a trained neural network receives the sample’s shape, dimensions, and refractive index as input parameters, outputting numerical parameters for precise control of spectral reflectance measurements on faceted gemstones, while simultaneously outputting strategic parameters for a fuzzy logic algorithm to determine the true color grade. The instrument is particularly suitable for multi-faceted gemstone testing due to its ability to dynamically adjust color data based on the sample’s size and cut. The color simulation was obtained using D65 illumination and a 10° observer. For accuracy, each sample was tested three times, and the average value was taken as the final result.

Specific gravity (SG) was determined by the hydrostatic weighing method, and the average of three measurements was taken. X-ray fluorescence (XRF) analysis was performed under vacuum using an EDX7000 X-ray fluorescence spectrometer manufactured by Shimadzu (Kyoto, Japan), employing the fundamental parameter (FP) method to obtain semi-quantitative compositional results. Analytical parameters were 15 kV, 1000 μA for Na-Sc, and 50 kV, 240 μA for Al-U. The FP method for quantitative analysis generates a “universal scale” equivalent to a standard, independent of standard samples, by comparing measured X-ray fluorescence spectral intensities with calculated spectra. The instrument is equipped with data analysis software (PCEDX Pro Analyzer, version 2.03) that automates the entire analytical workflow. Detection limits for the semi-quantitative instrument vary for each element. During the experimental phase, the detected spectra of each sample are thoroughly evaluated, and elemental content values with clearly identifiable spectral peaks are retained. While these values may be of minor magnitude, the results obtained are considered relatively reliable.

Micro-XRF analysis was performed at the Institute of Earth Sciences, China University of Geosciences (Beijing), using a Bruker M6 Jetstream mobile X-ray fluorescence (XRF) scanner (Bruker Nano GmbH, Berlin, Germany). The Bruker M6 Jetstream consists of a mobile measuring head equipped with a rhodium (Rh) target X-ray source (50 kV, 0.6 mA) and polycapillary optics, which can generate a spot size of less than 50 μm for Mo-Kα. The X-ray beam was perpendicular to the sample surface. The silicon drift detector (SDD) had an active area of 50 mm^2^ and an energy resolution of 145 eV for MnKα at 275 kcps. The scanner is capable of scanning the surface of a sample mounted on a motorized XY stage.

Ultraviolet-visible-near infrared (UV–Vis–NIR) spectra were measured using a GEM-3000 UV–Vis spectrophotometer by in-situ reflection method, manufactured by Biaoqi Detection Technology Co., Ltd. (Guangzhou, China). Each UV–Vis–NIR spectrum was recorded three times and averaged. The instrument is equipped with an integrating sphere that measures sample reflectance and converts it to transmittance or absorbance. Spectra were recorded over the range 350–1000 nm with 0.5 nm intervals and an integration time of 220 s. Infrared (IR) spectra were measured using a Bruker (Karlsruhe, Germany) Tensor 27 Fourier Transform Infrared Spectrometer at a resolution of 1 cm^−1^, equipped with a diffuse reflectance accessory (DRIFT) for in-situ reflectance testing of gemstone samples. IR spectra were obtained through 32 scans in reflectance mode (400–1500 cm^−1^) and transmission mode (7750–2200 cm^−1^). Raman spectra were measured using an HR-Evolution HORIBA laser Raman spectrometer (HORIBA Scientific, Longjumeau, France) with a 532 nm laser source, 3 s integration time, and two scans over the 100–1500 cm^−1^ acquisition range. All analyses were performed at the Gemmological Experimental Teaching Center, China University of Geosciences, and the Institute of Earth Sciences, China University of Geosciences (Beijing), Beijing, China.

## 3. Results

### 3.1. Standard Gemological Methods

**Figure 1:**
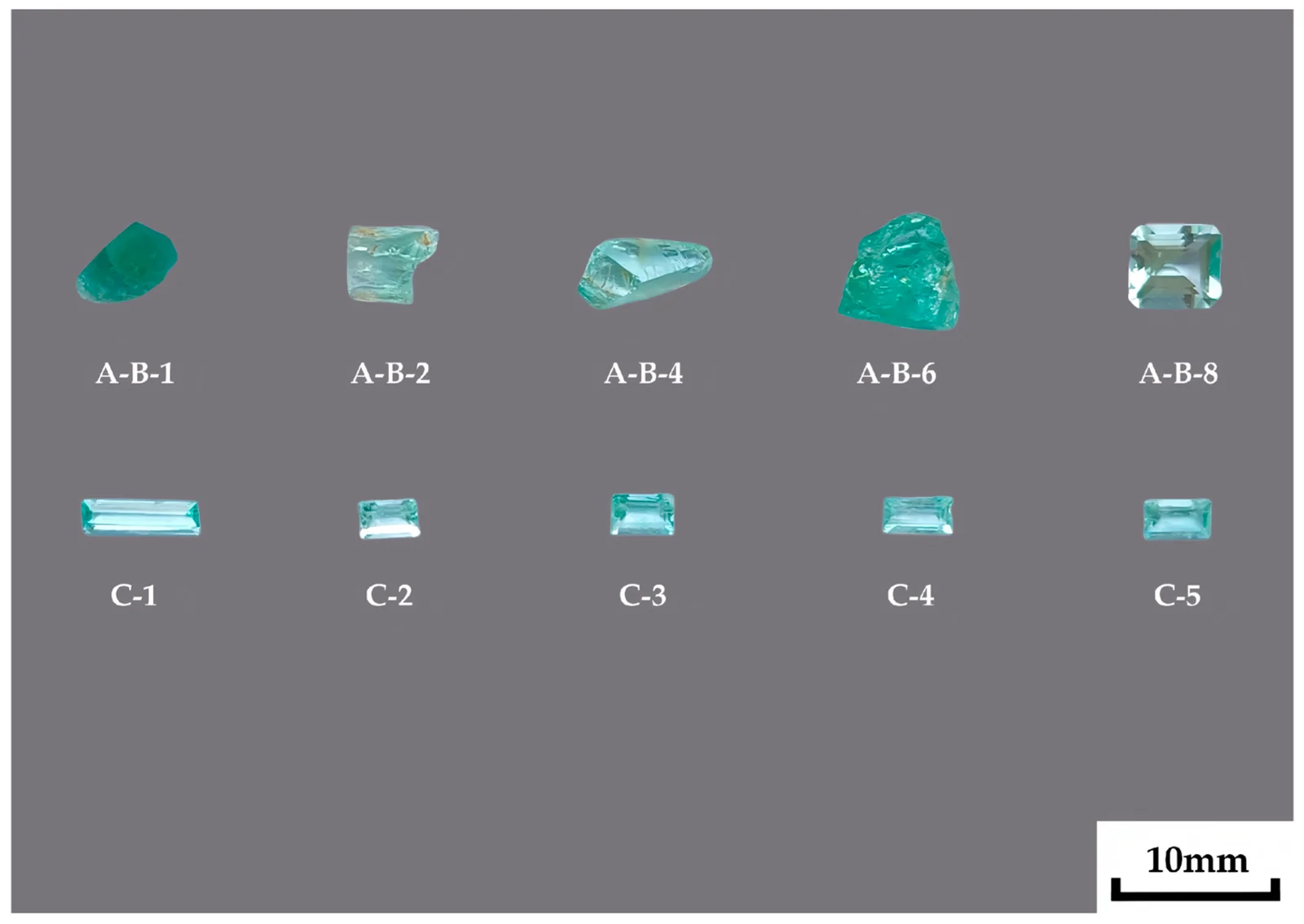
Photographs and sample numbers of the ten Nigerian blue-green beryl samples used for compositional and spectroscopic analyses.

All samples show vitreous luster and good transparency. Refractive index (RI) ranges between 1.567 and 1.583, birefringence (DR) between 0.006 and 0.008, and specific gravity (SG) between 2.6063 and 2.7174, all within the natural beryl range [10]. Samples are inert under UV, exhibit weak to moderate yellowish-green to bluish-green dichroism. Sample images are shown in Figure 1.

**Figure 2:**
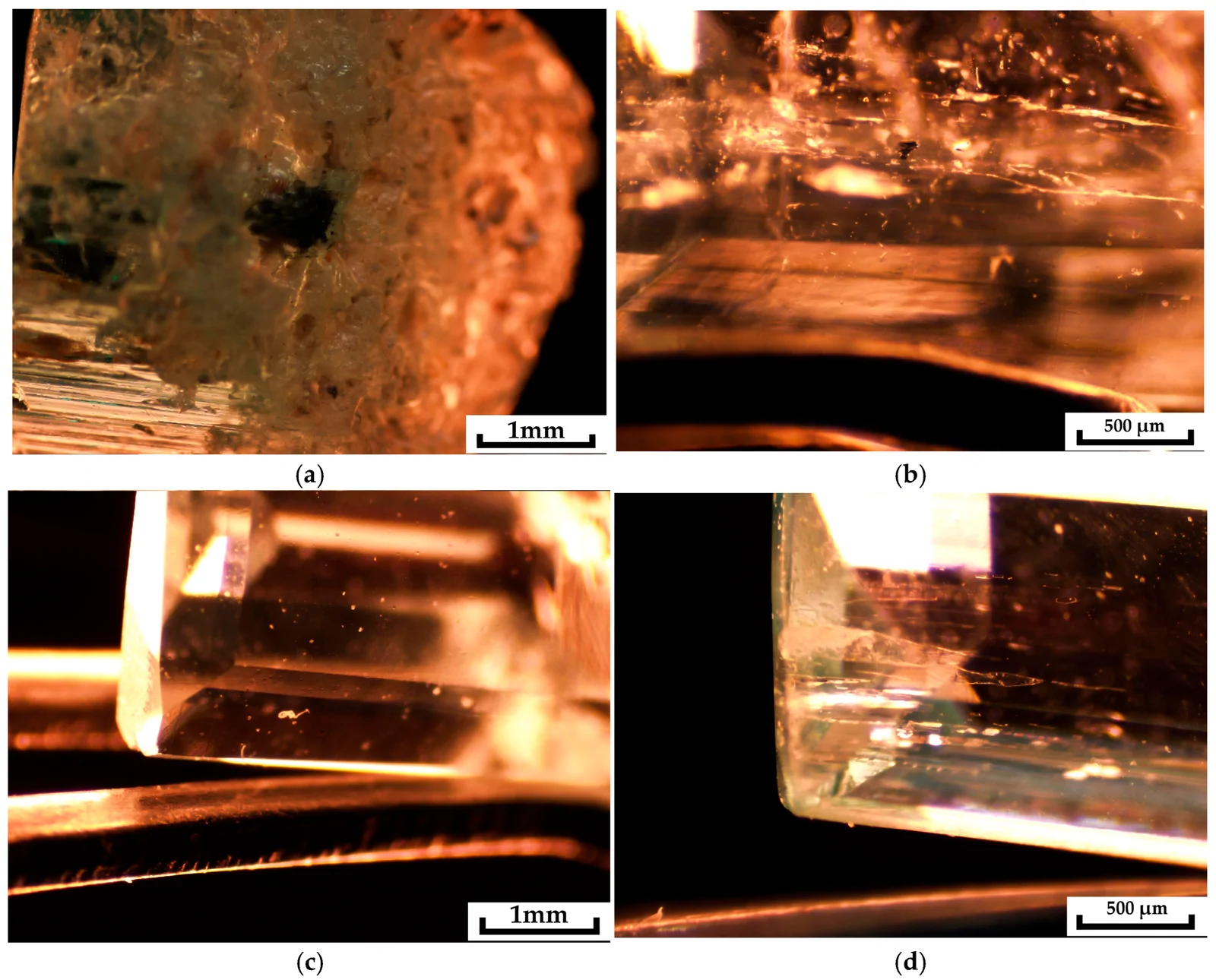
Photomicrographs of inclusions in selected samples: (**a**) black solid inclusion. (**b**) rain-like and jagged two-phase gas–liquid inclusions. (**c**) dot-like fluid inclusion. (**d**) rain-like two-phase gas–liquid inclusion.

Nigerian blue-green beryl contains various characteristic inclusions: solid inclusions are characterized by an assemblage of albite + fluorides + Fe-rich mica + ilmenite + monazite, rain-like inclusions are fine tubes parallel to the c-axis, similar to the raindrop-like inclusions in aquamarine; feather-like inclusions appear as veil-like healed fractures containing two- or three-phase fluids [7]; dot-like fluid inclusions are isolated or clustered, mainly two-phase gas–liquid, with CO_2_ as the dominant gas phase [7,9]. These inclusions collectively indicate a fluid-rich crystallization environment, microscopic images of inclusions are presented in Figure 2. Gemological details are in Table 1.

### 3.2. Color Quantization

Color was quantified using the CIE 1976 L*a*b* uniform color space, which is more scientifically accurate than visual observation, with parameters L* (lightness), a* (red-green), b* (yellow-blue), C* (chroma) and h° (hue angle, the range is 0–360°, with 0° corresponding to red, 90° to yellow, 180° to green, and 270° to blue). Results are presented in Table 2.

### 3.3. EDXRF Analysis

A total of 10 samples were included in this group. One random spot was measured on each sample, and the EDXRF data from all 10 spots are presented in Table 3. For sample A-B-1, the analytical spot was located on the outer surface. TFeO ranges between 0.117 and 1.782 wt.% (mean 0.6487), Cr_2_O_3_ ranges between 0.020 and 0.246 wt.% (mean 0.0758), V_2_O_5_ ranges between 0 and 0.122 wt.% (mean 0.034). TFeO is significantly higher than V_2_O_5_ and Cr_2_O_3_. Na was not detected, and Mg peaks were negligible.

**Figure 3:**
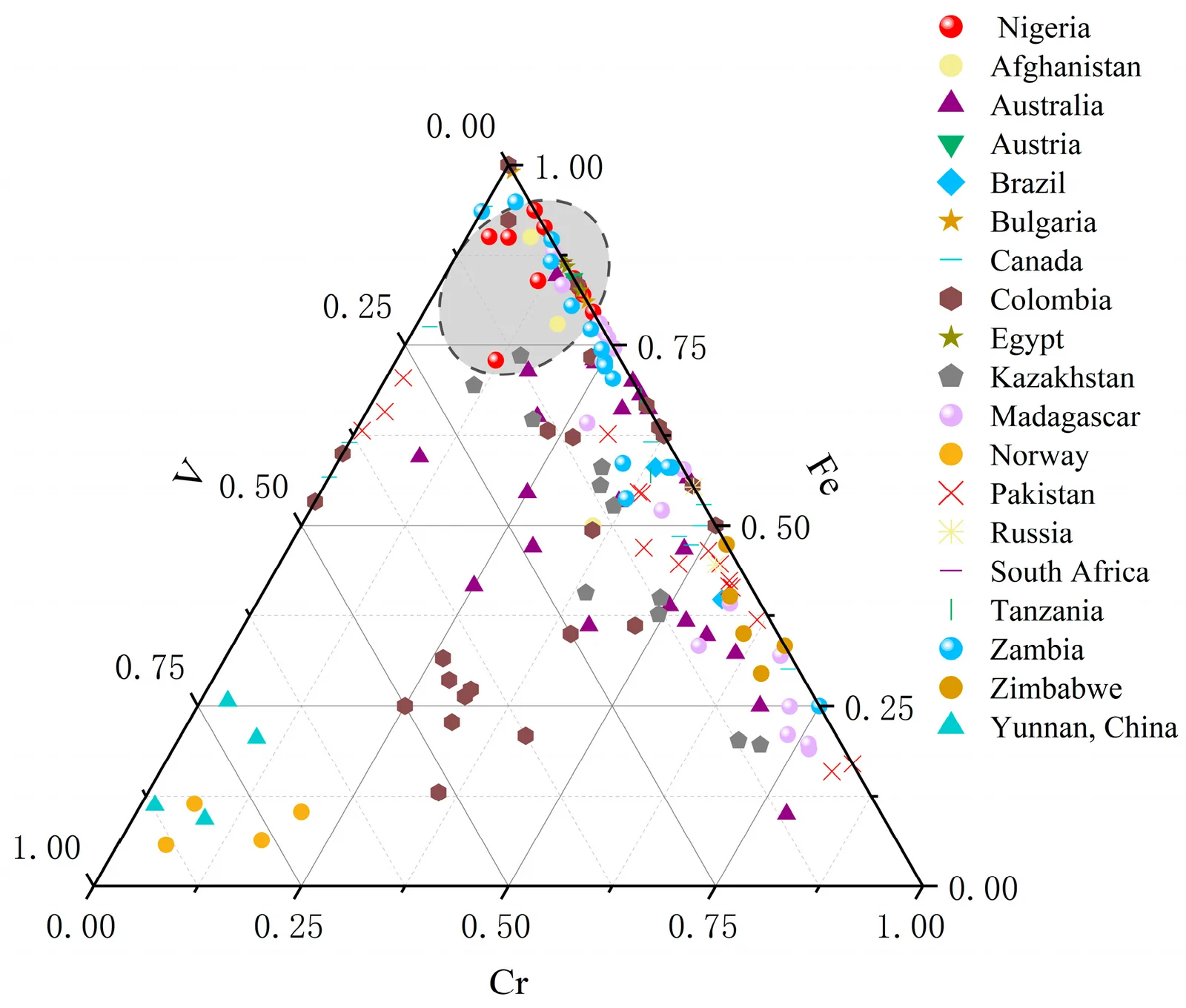
Standardized Fe–V–Cr ternary diagram for characterizing natural emeralds from Nigeria and other localities. Data for the Nigerian samples are from the present EDXRF analysis; comparative data are from references [5,12,13,14,15,16,17,18,19,20,21,22,23,24,25,26,27,28,29,30]. The three oxide-equivalent components were normalized to Fe + V + Cr = 100%. For samples with undetected V, the points are plotted on the V = 0 boundary for visualization; their positions do not indicate that the actual V content is zero. The gray shaded ellipse is only used to highlight the distribution of the Nigerian samples and has no statistical significance.

According to previous studies [4,11], emerald coloration is primarily attributed to Cr, V, and Fe. XRF data from the 10 Nigerian samples were combined with global emerald data for ternary diagram analysis (Figure 3). Nigerian beryls plot in the upper Fe-rich region of the diagram, with Fe proportions higher and Cr + V proportions lower than most emeralds. Their ternary projection is closest to Zambian and Egyptian samples. Nigerian samples show significant differences in Fe and V compared to most emeralds, and Cr contents are also lower. However, XRF only provides the total Fe content and cannot determine its specific valence state. Therefore, its valence-state information must be inferred in combination with UV–Vis–NIR spectra.

**Figure 4:**
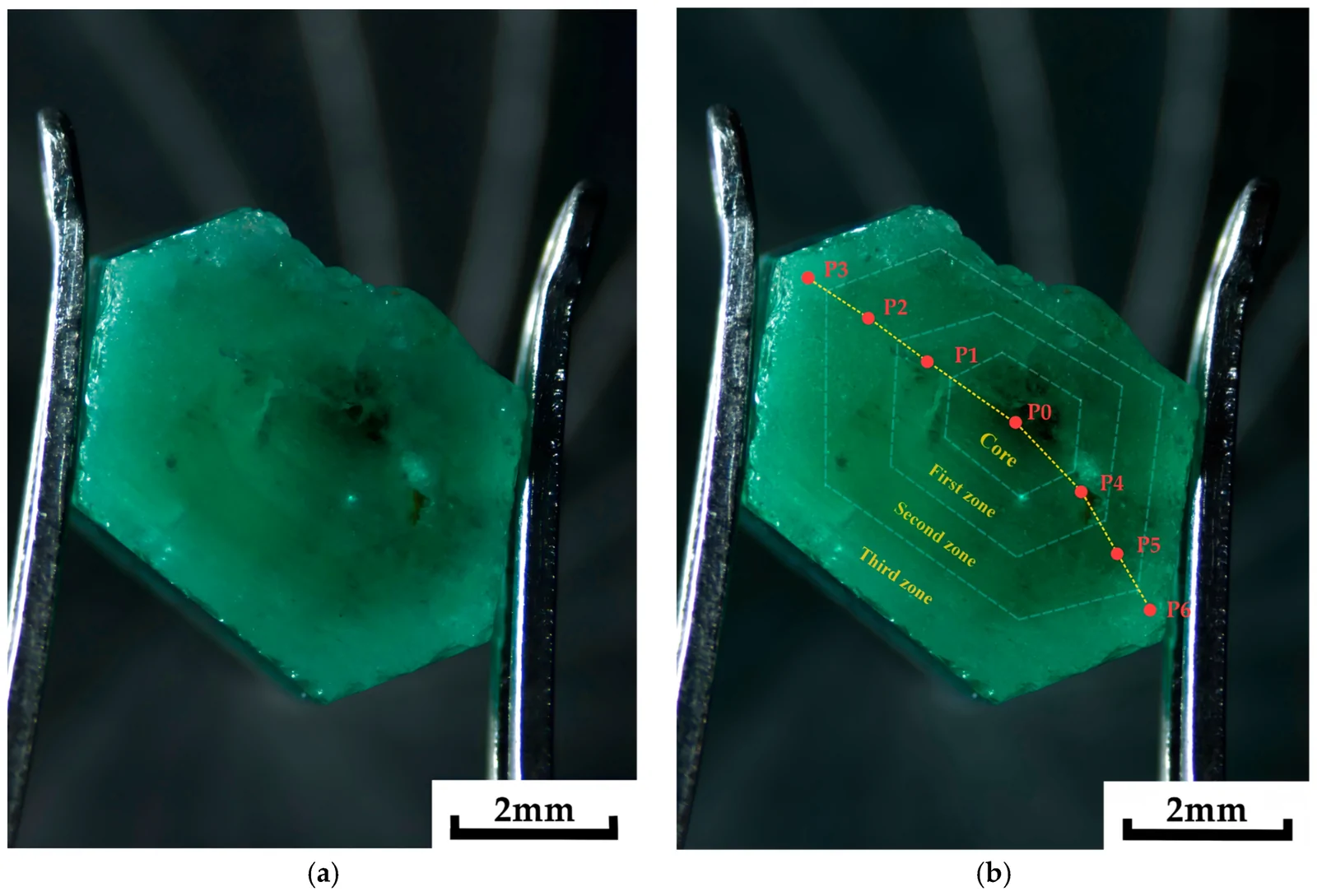
Original cross-section image of sample A-B-1 and EDXRF point selection positions on each zone: (**a**) Original crystal cross-section image of sample A-B-1. (**b**) Zonation map of the A-B-1 crystal cross-section and EDXRF analysis positions marked by red dots. The yellow dashed line connects the analysis positions, and the white dashed lines indicate the boundaries between zones.

Sample A-B-1, which exhibits a dark core and distinct color zoning, was selected for detailed point and micro-XRF analysis. The cross-section shows three concentric zones outward from the core: first zone (inner color zone), second zone (main color zone), and third zone (outer zone). Seven points (P0–P6) were analyzed (Table 4). Points P3–P6 form a transect across the section (Figure 4).

**Figure 5:**
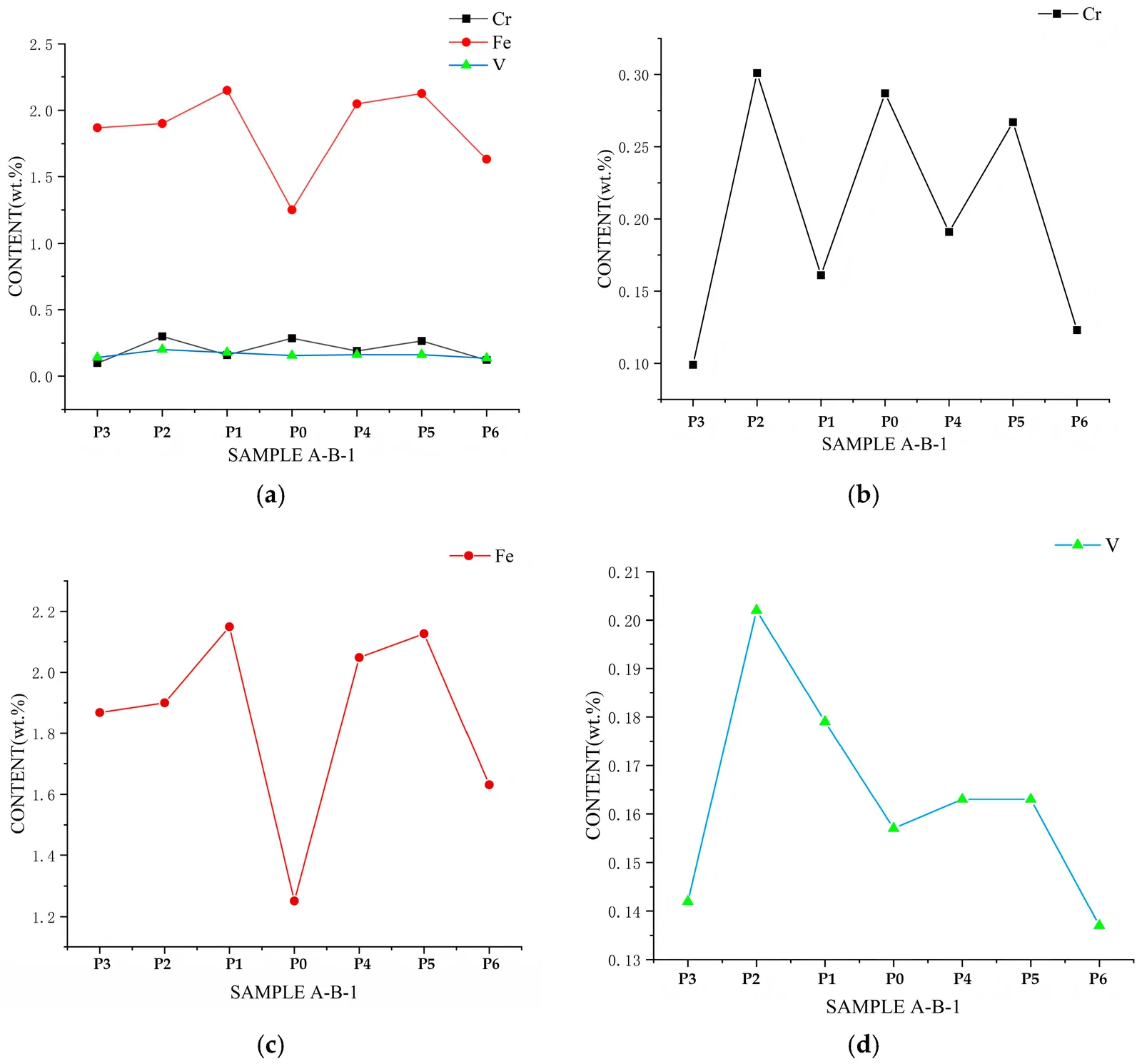
Variation of Relative Contents of Chromophoric Elements in the Crystal Cross-Section of Sample A-B-1: (**a**) Variation of Cr, Fe, and V contents in A-B-1 crystal cross-section. (**b**) Variation of Cr content in A-B-1 crystal cross-section. (**c**) Variation of Fe content in A-B-1 crystal cross-section. (**d**) Variation of V content in A-B-1 crystal cross-section.

As shown in Figure 5, the Cr content at the core and in the color zones of A-B-1 differs greatly from that in other parts of the crystal, reveals the complexity of element incorporation during beryl growth. The sequence—“Cr-rich core → Fe-rich first zone → Cr-re-enriched second zone → depletion in third zone”—defines a “Cr-Fe reverse relay zone” pattern.

**Figure 6:**
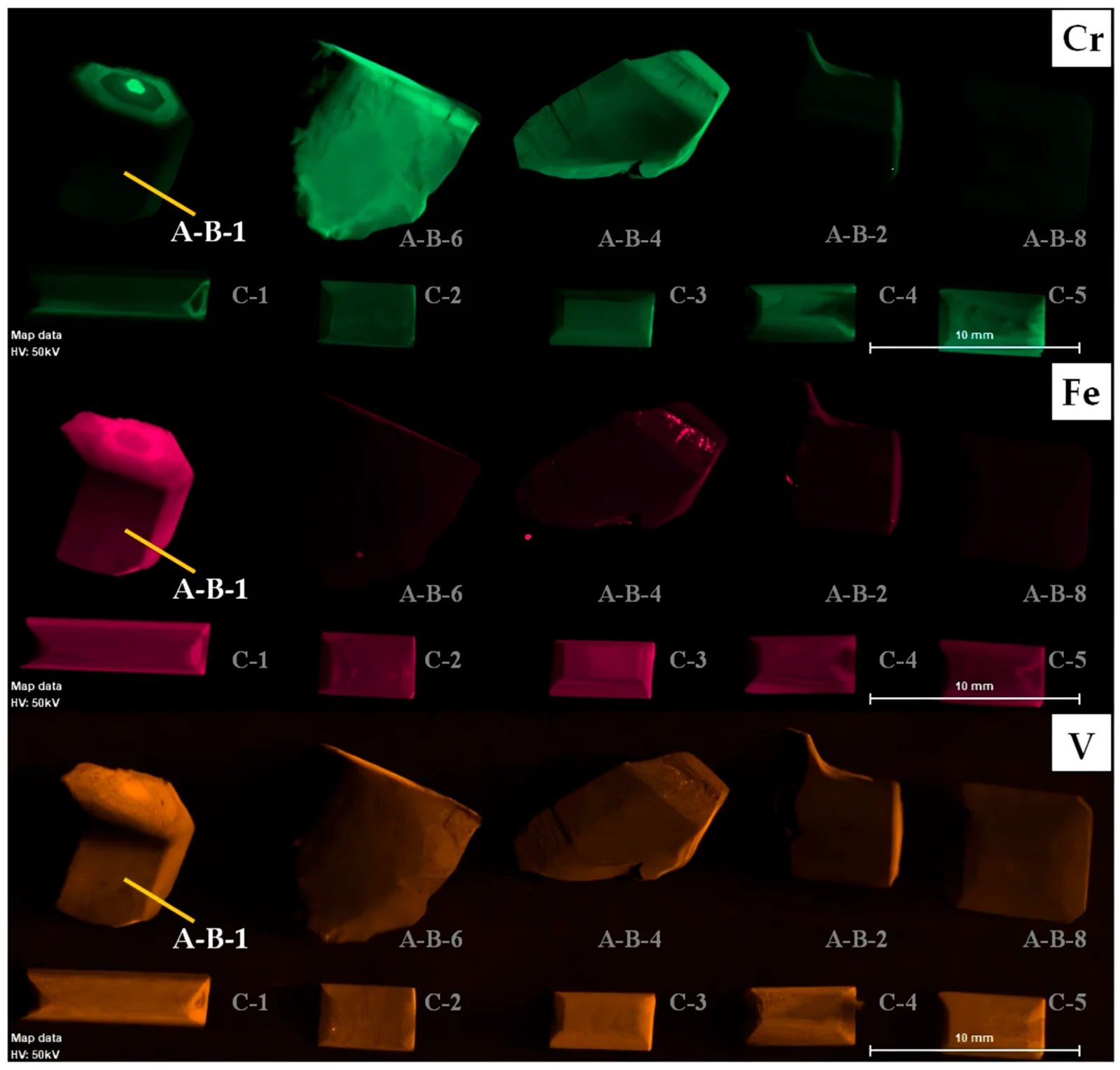
Micro-XRF elemental maps of Cr, Fe, and V for all samples. The sample order is the same as in Figure 1; A-B-1 is marked with an arrow. Intensities are normalized to the maximum counts of each element across all mapped samples, and the color scales are comparable among samples for the same element. The maps are therefore semi-quantitative and show relative elemental distributions rather than absolute concentrations. The colors used for different samples in this figure are intended solely for visual distinction and do not represent the actual colors of the chromophoric elements.

Micro-XRF (Figure 6) confirms the point-analysis results. The Micro-XRF instrument consists of a mobile measuring head that enables non-destructive elemental mapping analysis of small amounts of sample, even without pretreatment. It is equipped with a rhodium (Rh) target X-ray source (50 kV, 0.6 mA) and polycapillary optics, which can generate a spot size of less than 50 μm for Mo-Kα. The X-ray beam was perpendicular to the sample surface. For sample A-B-1, the analyzed surface was a crystal cross-section, whereas for the other samples, the analyzed surfaces were parallel to the c-axis. Micro-XRF showing strong Cr enrichment at the core, a Cr-depleted/Fe-enriched first zone, and less distinct V distribution due to its lower concentration. The distinct core-to-rim compositional zonation of A-B-1 defines a “Cr-Fe reverse relay zone” (Cr-rich core → Fe-rich first zone → Cr-re-enriched second zone → depleted third zone).

### 3.4. UV–Vis–NIR Spectroscopy

**Figure 7:**
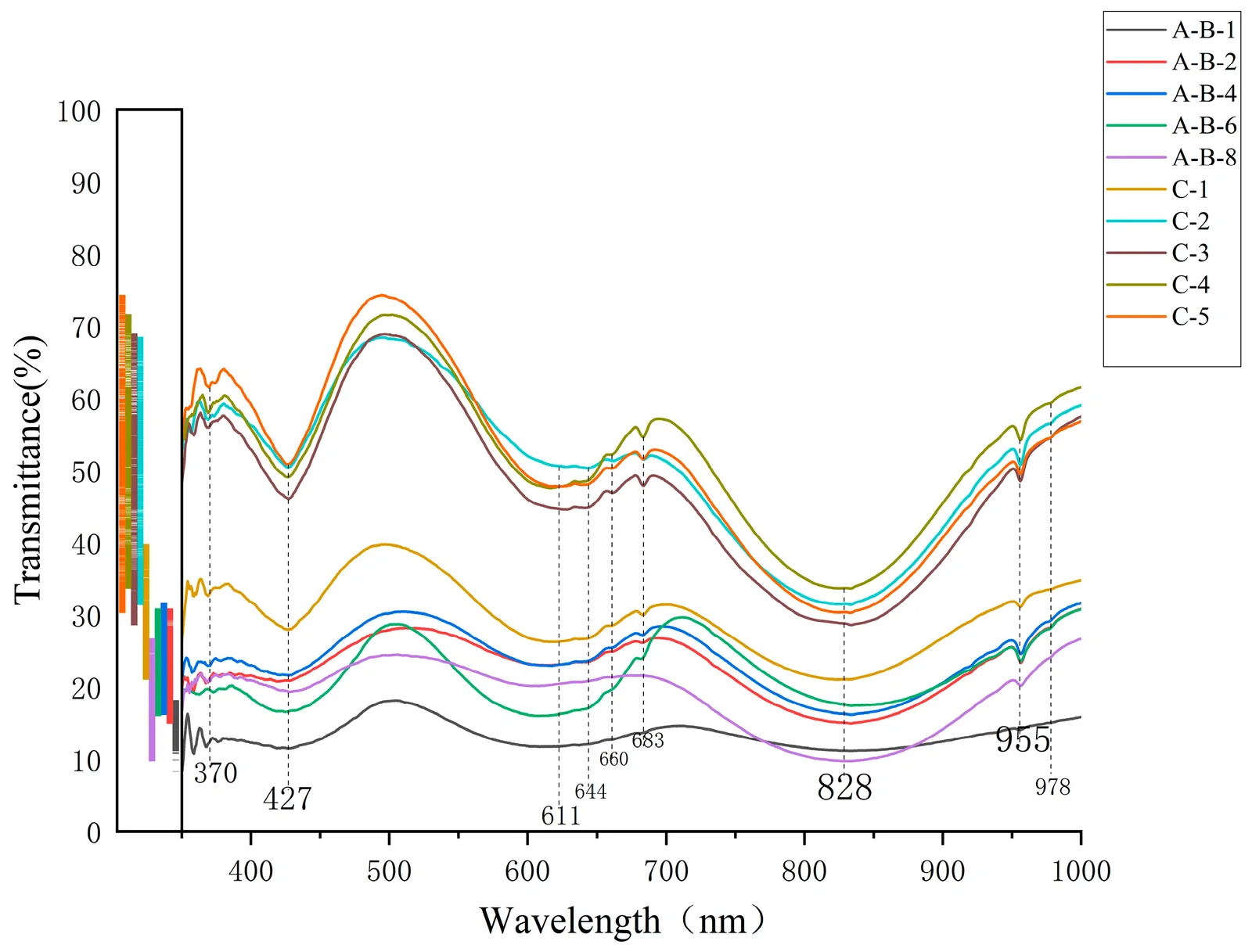
UV–Vis–NIR apparent transmittance spectra of all samples. The vertical axis is given in percent (%), and the same numerical scale is used for all samples. The spectra were recorded in reflectance mode and converted to apparent transmittance using the instrument software with a calibrated white reference.

The UV–Vis spectroscopic results and the EDXRF results corroborate each other. UV–Vis–NIR spectra at room temperature (Figure 7) show five main absorption bands after removing background artifacts below 350 nm: a sharp peak at 370 nm, a broad band at 427 nm, a broad band near 610 nm, a strong broad peak at 828 nm, and a narrow peak at 955 nm. The absorption band at 955 nm and the minor absorption peak at 978 nm are attributed to H_2_O stretching vibrations in the structural channels and d–d transitions of channel Fe^2+^ [31]. The 371 and 425 nm absorptions are Fe^3+^ spin-forbidden d–d transitions, with the 427 nm band weaker [3,32,33]. The 427 and 611 nm bands are mainly Cr^3+^/V^3+^ d–d transitions [34,35]. Schmetzer et al. [35] noted that Cr^3+^ and V^3+^ have similar band positions, but V^3+^ has stronger blue-violet absorption with a more extended edge, making their deconvolution difficult. The assignments of the absorption bands and narrow absorption peaks in the UV–Vis spectrum of beryl are listed in Table 5.

The 427 nm band has greater half-width and intensity than the 371 nm peak, indicating superposition of Fe^3+^ and Cr^3+^/V^3+^ absorptions. The 610 nm band is also composite: V^3+^/Cr^3+^ crystal-field transitions plus Fe^2+^–Fe^3+^ IVCT [36,37]. The 828 nm ultra-broad band is attributed to two Fe^2+^ contributions: spin-allowed d–d transitions of six-coordinated Fe^2+^ in octahedral Al sites, and electronic transitions of channel Fe^2+^. The 828 nm absorption band, assigned to Fe^2+^ d–d transitions, is the strongest feature, indicating a significant Fe^2+^ contribution. The 427 nm Fe^3+^ band and the 611 nm composite band (Cr^3+^/V^3+^ plus Fe^2+^–Fe^3+^ IVCT) are weaker but comparable in intensity, suggesting that Fe^3+^ is also present in appreciable amounts and that Fe^2+^–Fe^3+^ IVCT pairs are abundant. These features collectively support a multi-ion synergistic coloration model in which Fe^2+^ dominates the blue component, Fe^3+^ contributes a yellow component, and their IVCT interaction enhances the blue-green hue [38,39,40]. These multi-ion absorptions create a “transmission window” in the blue-green region, forming the spectroscopic basis for the blue-green hue.

### 3.5. FTIR Spectroscopy

**Figure 8:**
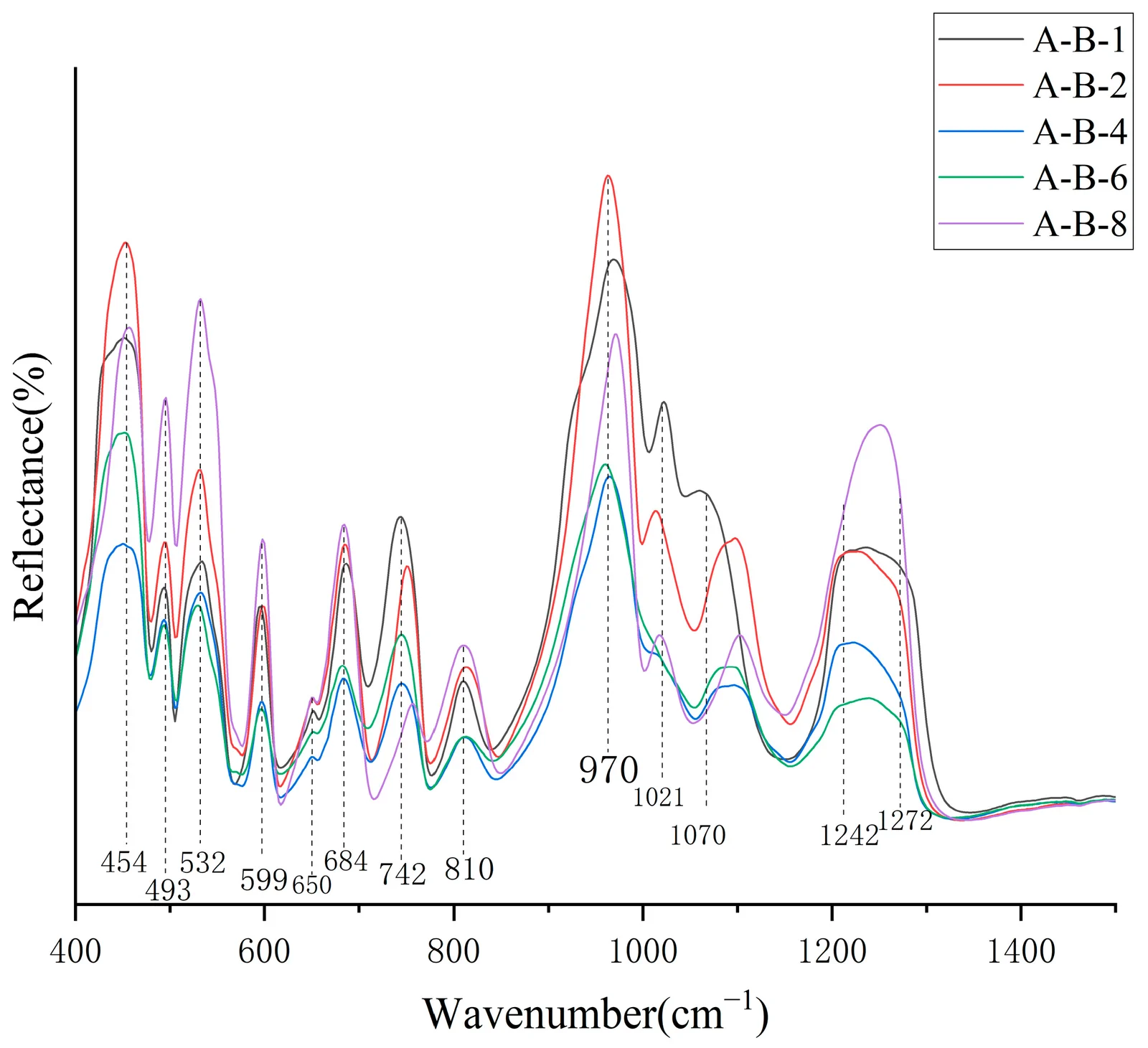
IR reflectance spectra of selected samples with E⊥c (400–1500 cm^−1^).

IR spectra were collected in reflectance (400–1400 cm^−1^) and transmission (2000–8000 cm^−1^) modes perpendicular to the c-axis. Reflectance spectra of selected representative samples are shown in Figure 8. Reflectance spectra show consistent absorption features. The 1070 cm^−1^ Si–O stretching peak is sensitive to octahedral Al-site substitution: replacement of Al^3+^ by Fe^2+^/Mg^2+^ causes local charge imbalance, inducing electron cloud rearrangement in adjacent Be–O/Si–O bonds that increases the Si–O force constant and shifts the stretching frequency to higher wavenumbers by 10–30 cm^−1^ [2]. Samples A-B-2, A-B-4, A-B-6, and A-B-8 show stronger and higher-wavenumber-shifted 1070 cm^−1^ peaks and weaker/displayed 1242 cm^−1^ peaks, consistent with their lower TFe contents [9,40].

**Figure 9:**
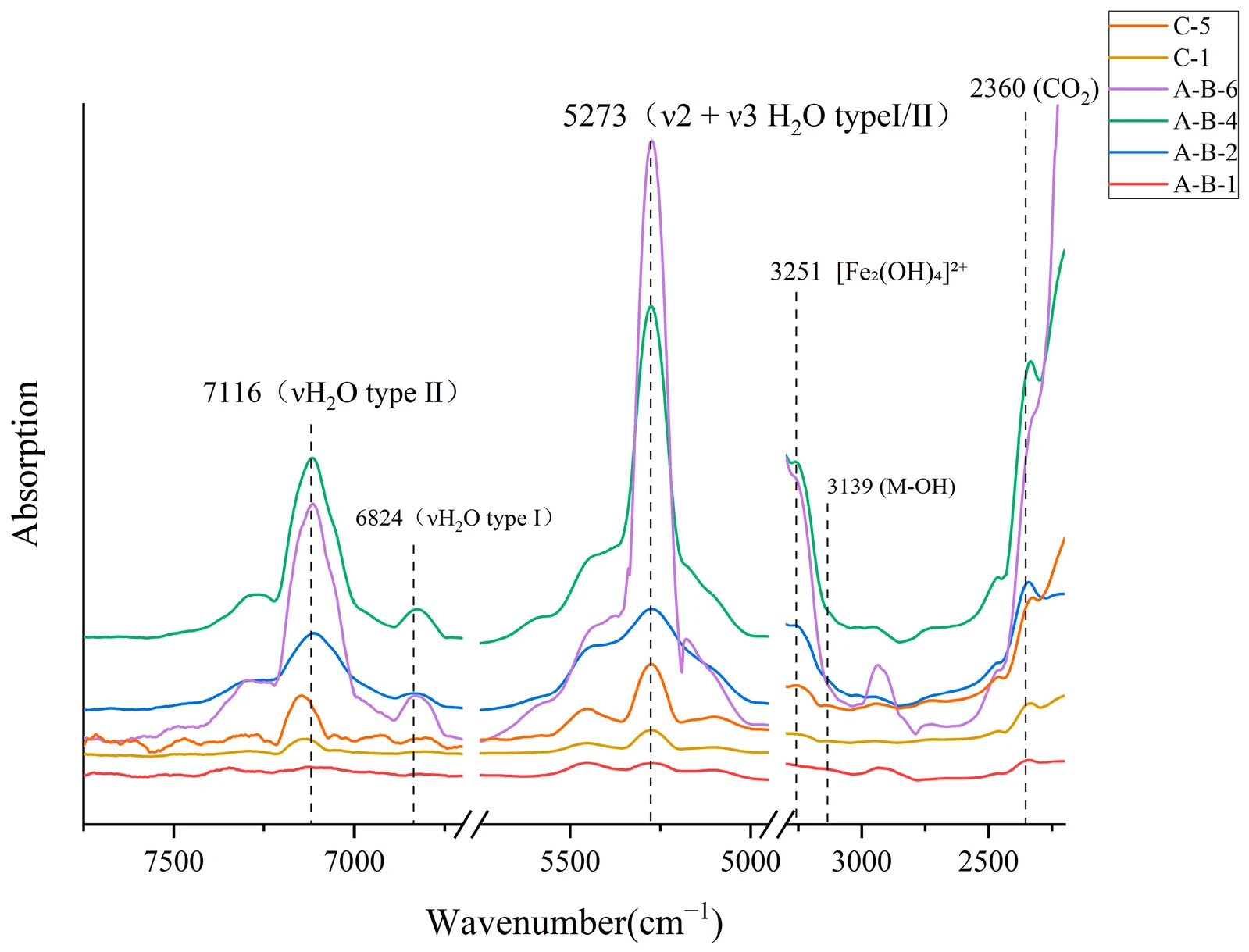
IR transmission spectra of selected samples with E⊥c (2200–7750 cm^−1^).

Transmission spectra (Figure 9) show absorptions from structural channel groups: 2000–3500 cm^−1^ from hydrated ions, alkali metal complexes, and CO_2_; 3500–8000 cm^−1^ from H_2_O stretching. All samples show a sharp 5273 cm^−1^ peak. Type I H_2_O shows dominant 7116 cm^−1^ (ν_3_ asymmetric stretching) with a weak 6824 cm^−1^ peak; Type II water at 7060 cm^−1^ is very weak or absent in some samples [41,42,43,44].

### 3.6. Raman Spectroscopy

Raman spectra were collected perpendicular to the c-axis using a 532 nm laser over the range 100–1500 cm^−1^. Because the laser spot may simultaneously excite both the beryl host and inclusions, the inclusion spectra usually contain both host beryl peaks and characteristic inclusion peaks. Therefore, the Raman results are discussed separately for the beryl matrix and fluid inclusions.

**Figure 10:**
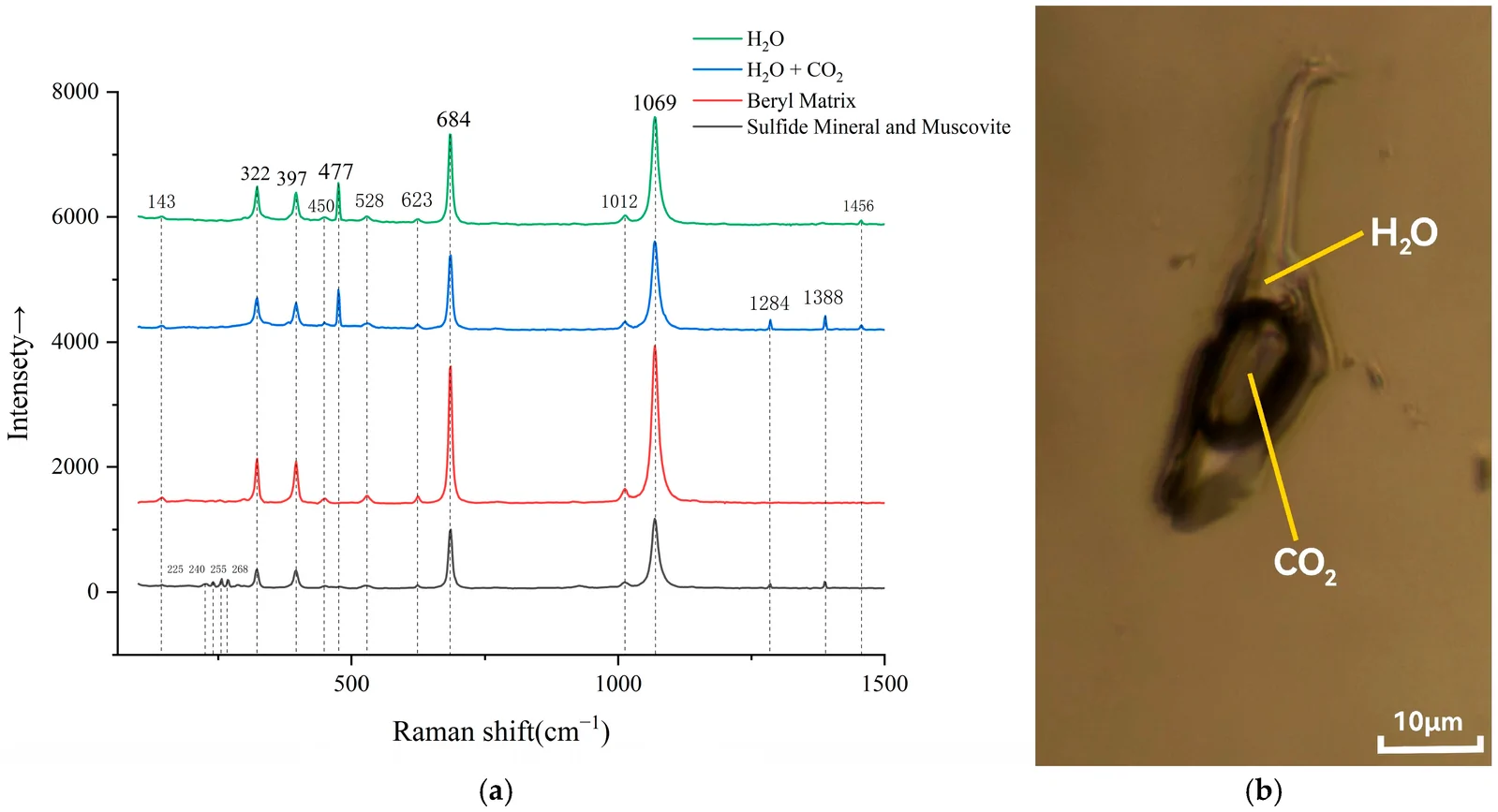
Raman spectra and inclusions: (**a**) Raman spectra of beryl matrix and inclusions. (**b**) Fluid two-phase inclusions in sample C-5.

As shown in Figure 10a, the beryl matrix spectra exhibit consistent peak positions at 322, 397, 684, 1012, and 1069 cm^−1^, which are assigned to six-membered ring deformation, Al–O vibration, Be–O stretching, and Si–O stretching, respectively [41,42,45]. These peaks appear in all samples, confirming the beryl structure. Minor variations in peak position and intensity may reflect minor substitution of Fe, Cr, and V at the Al site; however, because the degree of substitution is low, the shifts are small [42,43].

In the H_2_O + CO_2_ spectra and sample C-5, the CO_2_ Fermi resonance doublet of fluid inclusions at 1284 and 1388 cm^−1^ is clearly observed (Figure 10a), indicating that the fluid inclusions contain CO_2_. The H_2_O-bearing spectra also show host beryl peaks. The coexistence of H_2_O and CO_2_ in two-phase inclusions (Figure 10b) indicates that the Nigerian beryl crystallized in a carbon-rich, aqueous fluid environment. This is consistent with the CO_2_ absorption at 2360 cm^−1^ in the IR spectra [44].

## 4. Discussion

The preceding analyses have systematically documented the gemological, chemical, and spectroscopic characteristics of Nigerian blue-green beryl, revealing distinctive chemical fingerprints from macroscopic chromaticity to micron-scale elemental distribution. This section evaluates the chromaticity effects of V^3+^ incorporation and TFeO/(Cr_2_O_3_ + V_2_O_5_) ratio, and integrates these observations into a multi-ion synergistic coloration model.

**Figure 11:**
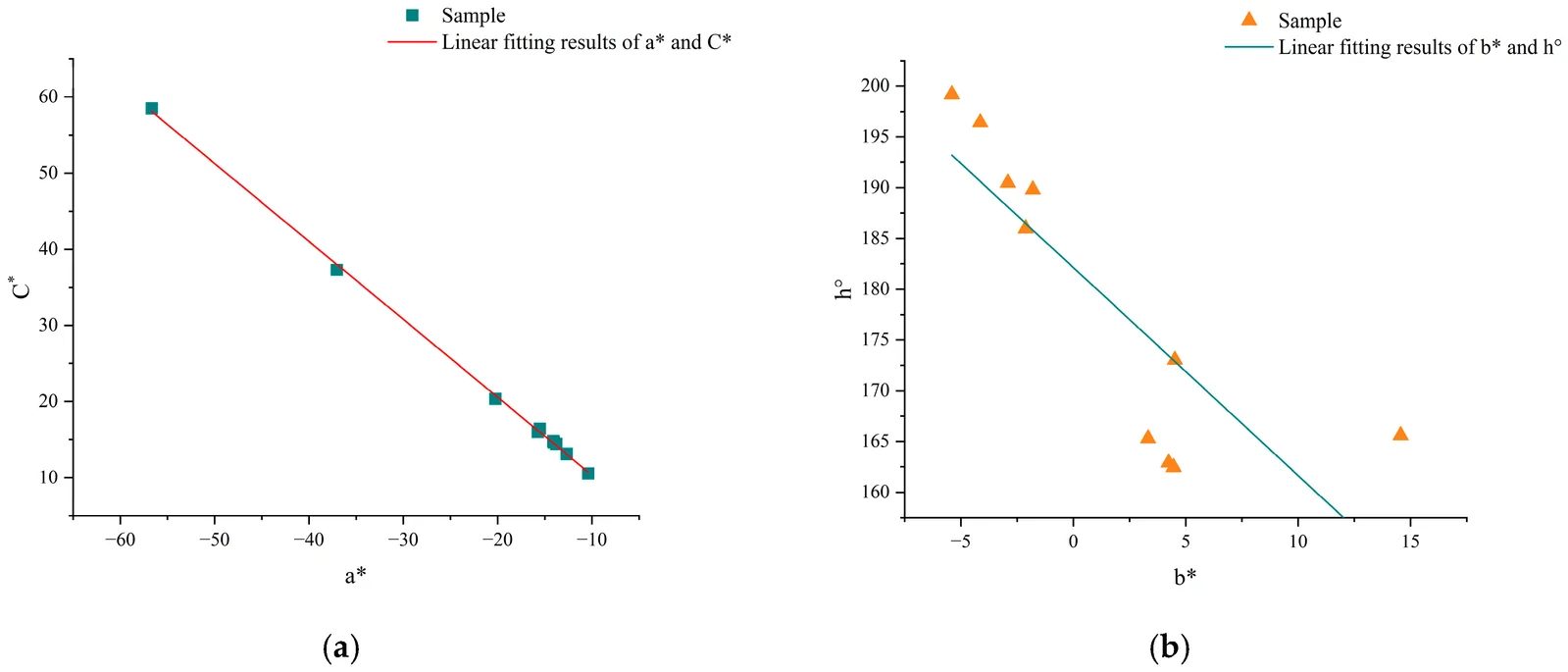
Linear fitting results of the chromaticity parameters of all samples: (**a**) Linear fitting results of a and C*. (**b**) Linear fitting results of b* and h°.

The color parameters of the samples fall within the following ranges: L* ∈ 34.97–95.42, a* ∈ −56.6 to −10.40, b* ∈ −5.40 to 14.56, C* ∈ 10.55–58.50, h* ∈ 162.5–199.2°, indicating that the samples are situated in the blue-green hue range with medium-to-high lightness and low chroma (Table 2). C* shows a strong negative correlation with a*, and h shows a strong negative correlation with b* (Figure 11): lighter green tones have lower saturation, and more yellow tones have smaller hue angles.

Three-Dimensional Color Difference (ΔE*_ab_): The three-dimensional color difference incorporates three dimensions—lightness L*, the red-green chromaticity coordinate a*, and the yellow-blue chromaticity coordinate b*—and is the standard total color difference formula in CIELAB. Its formula is:

(1) $$\Delta E_{\mathrm{ab}}^{*}=\sqrt{{\left(\Delta L^{*}\right)}^{2}+{\left(\Delta a^{*}\right)}^{2}+{\left(\Delta b^{*}\right)}^{2}}$$

Two-Dimensional Color Difference (Δab*): If only the chromaticity difference on the a*–b* plane is considered while ignoring lightness L*, the Euclidean distance between two points on the chromaticity plane is given by:

(2) $$\Delta ab^{*}=\sqrt{{\left(\Delta a^{*}\right)}^{2}+{\left(\Delta b^{*}\right)}^{2}}$$

Chroma Difference (ΔC*ab): The chroma difference is an index in the CIELAB color space used to quantify the difference in saturation (vividness) between two colors. It reflects only changes in color “intensity/vividness” and does not include differences in lightness (L*) or hue (h). Its formula is:

(3) $$\Delta C_{\mathrm{ab}}^{*}=C_{1}^{*}-C_{2}^{*}=\sqrt{{\left(a_{1}^{*}\right)}^{2}+{\left(b_{1}^{*}\right)}^{2}}-\sqrt{{\left(a_{2}^{*}\right)}^{2}+{\left(b_{2}^{*}\right)}^{2}}$$

**Figure 12:**
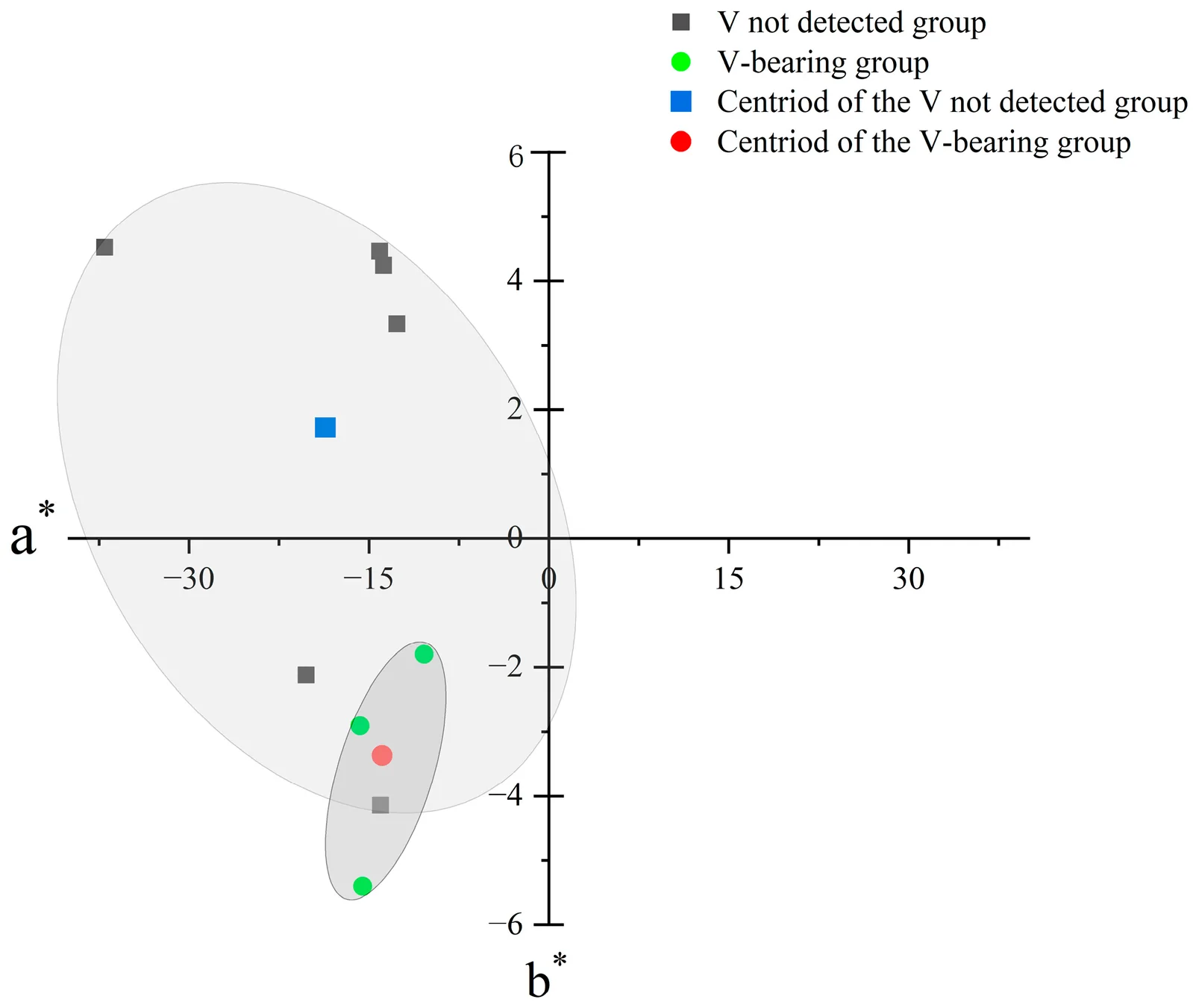
Chromaticity ranges and mean centroids of the “V-bearing” and “V not detected” groups. The light gray ellipse represents the simulated distribution region of the “V not detected” group, and the dark gray ellipse represents the simulated distribution region of the “V-bearing” group.

Because undetected vanadium indicates extremely low V content, such samples are hereafter referred to as “V not detected”. Samples were divided into “V not detected” (number of samples = 6: A-B-6, A-B-4, A-B-2, A-B-8, C-3, C-4) and “V-bearing” groups. The “V-bearing” group originally included A-B-1, C-1, C-2, and C-5. A-B-1 was excluded as an extreme outlier: its cross-sectional distribution of color and Cr, Fe, and V contents is highly heterogeneous, with a* = −56.66, far outside the range of the other V-bearing samples (−15.74 to −15.53), deviating by more than 1.49 standard deviations from the group mean, and it is the only sample with Cr/V ≈ 1.00. Its inclusion would bias the V-bearing centroid toward green. Including A-B-1, the V-bearing centroid is (L* = 62.41, a* = −24.58, b* = 1.11); excluding it, the refined V-bearing group (n = 3: C-1, C-2, C-5) has a centroid at (L* = 71.55, a* = −13.89, b* = −3.37), while the “V not detected” group centroid is (L* = 69.35, a* = −18.65, b* = 1.72). Notably, the direction of the a* shift relative to the “V not detected” group depends on whether A-B-1 is included: with A-B-1, the V-bearing group has a more negative a* (greener); without it, the V-bearing group has a more positive a* (less green, more blue-green). These results are summarized in Table 6. The three-dimensional color difference (ΔE*_ab_ = 7.31) exceeds the commonly cited perceptibility threshold of 2.3 [45], while the two-dimensional chromaticity difference (Δab* = 6.97) also indicates a substantial shift in hue and saturation (Figure 12).

When A-B-1 is excluded, the V-bearing centroid shows a* positive shift (green weakening, +4.76) and b* negative shift (blue deepening, −5.09), indicating a more blue-green average color. This appears contrary to the expectation that V^3+^ strong blue-violet absorption should produce yellowish-green [33], but reflects the dominant influence of Fe^2+^–Fe^3+^ IVCT red-region absorption [35], which visually imposes a strong blue tone. The remaining V-bearing samples (C-1, C-2, C-5) have high Fe contents (0.59–1.78 wt%) and negative b* values (−2.91 to −5.40), confirming that Fe IVCT dominates the blue tone in V-bearing samples.

**Figure 13:**
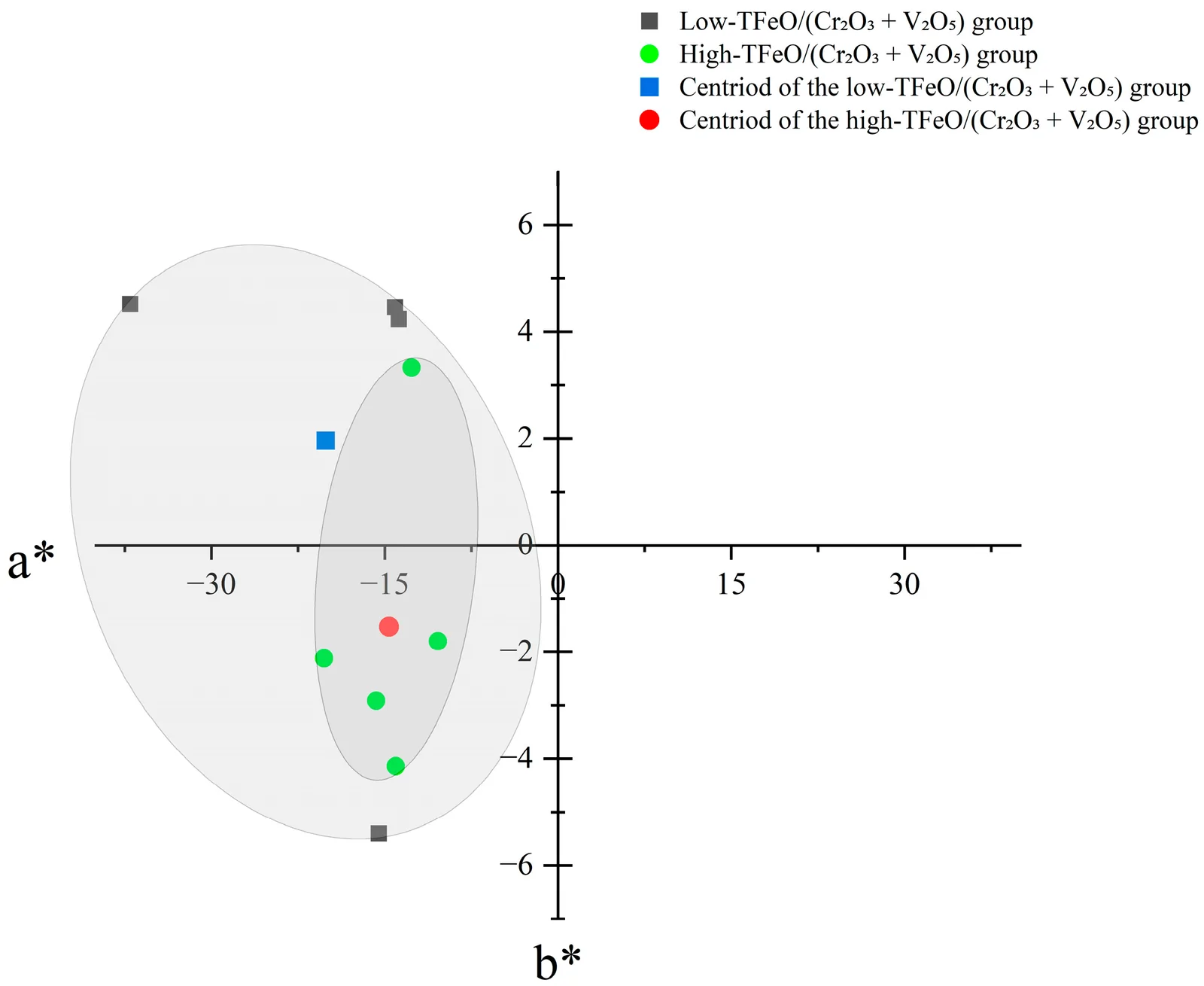
Chromaticity ranges and mean centroids of the “high-TFeO/(Cr_2_O_3_ + V_2_O_5_)” and “low-TFeO/(Cr_2_O_3_ + V_2_O_5_)” groups. The light gray ellipse represents the simulated distribution region of the “low-TFeO/(Cr_2_O_3_ + V_2_O_5_)” group, and the dark gray ellipse represents the simulated distribution region of the “high-TFeO/(Cr_2_O_3_ + V_2_O_5_)” group.

To distinguish TFeO from Cr_2_O_3_ + V_2_O_5_ contributions, the TFeO/(Cr_2_O_3_ + V_2_O_5_) ratio was introduced. This ratio ranges from 2.69 (C-5) to 14.86 (C-3). To balance the sample sizes between the high- and low-ratio groups as much as possible, using TFeO/(Cr_2_O_3_ + V_2_O_5_) = 5.0 as the boundary, as listed in Table 7, corresponding to a natural break in the data distribution. This value, used as the grouping criterion for high and low ratios, is applicable only to the present batch of experimental samples and should not be generalized to all blue-green beryl samples. The nine samples (excluding A-B-1) were divided into a low-ratio group (n = 4: A-B-2, A-B-4, A-B-6, C-5; mean 4.01) and a high-ratio group (n = 5: C-1, C-2, C-3, C-4, A-B-8; mean 8.99). Compared with the low-ratio group (L* = 59.35, a* = −20.12, b* = 1.96), the high-ratio group centroid (L* = 78.67, a* = −14.62, b* = −1.53) exhibits a positive shift in a* (+5.50), a negative shift in b* (−3.49), an increase in lightness (+19.32), and a decrease in chroma (−5.82). This corresponds to a hue shift from yellowish-green (174.4°) to blue-green (186.0°). The three-dimensional color difference is 20.39, with lightness contributing approximately 90%, whereas the two-dimensional chromaticity difference of 6.51 remains perceptually significant (Figure 13).

The TFeO/(Cr_2_O_3_ + V_2_O_5_) ratio and V-bearing/V not detected grouping have similar 2D chromaticity discrimination (~6.5–7.0), but TFeO/(Cr_2_O_3_ + V_2_O_5_) has much stronger lightness discrimination (ΔL* = 19.32). This confirms that absolute Fe content strongly affects lightness, consistent with Wang et al. [40] finding that higher total transition metal content lowers lightness. The high-ratio group contains A-B-8 (near-colorless, Fe only 0.211 wt%), which anomalously raises group lightness, indicating that the lightness indication of the TFeO/(Cr_2_O_3_ + V_2_O_5_) ratio is constrained by absolute Fe^2+^ content.

Both grouping schemes show consistent displacement directions (a* positive, b* negative), demonstrating that TFeO enrichment over Cr_2_O_3_ + V_2_O_5_ is the core driver of the yellowish-green to blue-green hue shift, while V provides only a constant background overlay. The TFeO/(Cr_2_O_3_ + V_2_O_5_) ratio thus serves as an effective hue predictor: higher ratios (Fe-enriched) correlate with more blue-green (b* negative); lower ratios (Cr_2_O_3_ + V_2_O_5_-enriched) correlate with more yellowish-green (b* positive). This is consistent with Fe^2+^–Fe^3+^ IVCT contributing to blue and Cr^3+^/V^3+^ contributing to yellow-green. At the micro-zoning scale (Section 3.3), Fe drives color variation while V remains relatively uniform, further confirming that Fe is the main driver of hue variability that distinguishes this material from emeralds of other origins, with V as a yellowish-tone contributor.

XRF data show that Ni, Mn are present only in trace amounts (NiO ≤ 0.018 wt%, MnO ≤ 0.165 wt%, and TiO_2_ contents are also low, with most samples below the detection limit). Previous studies have also noted that the color changes of their samples are controlled by the valence conversion and site occupancy changes of Fe^2+^/Fe^3+^, without mentioning any contribution from Ti, and in the trace-element and colorimetric data of this sample group, The elevated TiO_2_ contents in C-2 (1.149 wt%) and C-3 (0.747 wt%) are attributed to Ti-rich micro-inclusions (e.g., rutile, ilmenite, or Ti-bearing mica). XRF provides bulk elemental concentrations and may incorporate contributions from such inclusions. The absence of a systematic color difference between these samples and low-Ti samples suggests that Ti does not act as a significant chromophore in this material. Under high-Fe conditions, Mn ions do not participate in producing the blue-green hue, and no characteristic Mn absorption is present in their UV–Vis spectra. Ni^2+^ may influence coloration only in special copper-colored varieties. Previous studies [1,3,9,40,46,47] have excluded these elements as significant chromophores in this beryl type, confirming that the blue-green color is purely controlled by Cr, V, and Fe.

The Raman results cannot directly prove the origin of the blue-green color, because color-causing ions such as Fe, Cr, and V reside in the beryl lattice rather than in inclusions. However, CO_2_-bearing fluid inclusions indicate a carbon-rich fluid environment that may have influenced the Fe^2+^/Fe^3+^ ratio and the valence states of Cr and V during crystallization, thereby indirectly affecting color. Therefore, the Raman data mainly constrain the crystallization environment and inclusion characteristics, whereas the color mechanism is still controlled by multi-ion synergistic interactions among color-causing ions in the beryl structure.

The “Cr–Fe reverse relay zone” in the cross-section of A-B-1 may indicate an early Cr-rich fluid pulse followed by Fe–V-rich perturbation. This may suggest a possible early Cr-rich stable crystallization environment, followed by a mid-stage Fe–V-rich fluid perturbation. Zhang and Lan [48] noted that Cr-rich early fluids could produce dark cores; Rondeau et al. [49] showed that Cr distribution lacks global uniformity. Zheng et al. [4] described zigzag, wavy, or conical color zones in hydrothermal emeralds, and Rondeau et al. [49] reported strong color zones parallel to the basal plane, possibly indicating that transient Cr-rich pulses may cause localized core enrichment. Wu et al. [2] summarized that isomorphic substitution causes lattice distortion, and Zhang and Lan [48] noted that microfractures and inclusion margins are high-energy sites for foreign ion capture. Wood and Nassau [3] stated that pure Fe does not enter the lattice but forms channel iron; the high-Fe environment in the first zone of A-B-1 appears consistent with this model. The Cr enrichment at the core may thus result from multiple factors: early Cr-rich fluids, pulsed compositional fluctuations, selective capture by crystal defects, and the unique valence-state coordination of beryl. The coupling between this color zoning distribution and color appearance may add plausibility to the hypothesis proposed in this study: high-Cr/low-Fe zones may yield pure green, whereas high-Fe/low-Cr zones may introduce blue tones.

## 5. Conclusions

Nigerian blue-green beryls are characterized by low alkali and magnesium contents, minimal impurity elements, and very low Al substitution, resulting in relatively low RI and SG values, with iron (Fe^2+^, Fe^3+^, and their IVCT) and chromium (Cr^3+^) as the main chromophores jointly producing the yellowish-green to blue-green hue range. Spectroscopically, the 427 and 610 nm UV–Vis–NIR absorptions are characteristic of V^3+^ (and minor Cr^3+^), with Fe^2+^–Fe^3+^ IVCT superimposed on the 610 nm band; the stronger blue-violet absorption of V^3+^ imparts a yellowish-green base tone, which combines with the light blue from Fe IVCT to generate the distinctive “electro-optical” green color. Quantitative chromaticity analysis independently confirms that V’s yellowish-green contribution is insufficient to offset the strong blueward shift imposed by Fe^2+^, and that Fe enrichment relative to Cr^3+^ + V^3+^ is the key factor driving the hue from yellowish-green to blue-green (ΔEab = 7.31 between “V-bearing” and “V not detected” groups; Δab* = 6.51 between “high-TFeO/(Cr_2_O_3_ + V_2_O_5_)” and “low-TFeO/(Cr_2_O_3_ + V_2_O_5_)” groups). The “Cr-Fe reverse relay zone” revealed by micro-XRF further demonstrates that Cr and Fe independently define the color appearance—high-Cr/low-Fe zones yield pure green, high-Fe/low-Cr zones introduce blue tones, and V acts as a yellowish auxiliary modulator. The spectroscopic results and trace-element results collectively support this conclusion. By integrating trace-element, spectroscopic, and chromaticity constraints, this study provides new perspectives and insights into the coloration mechanism of Nigerian blue-green beryl, a highly sought-after gemological material. Whether this conclusion is generally applicable to emeralds or aquamarines of other color grades remains to be further investigated. Future collection and testing of data from a series of blue-green beryl samples spanning a chromaticity gradient across different localities would further verify the specific influence mechanism of chromophoric elements on the chromaticity of beryl.

## Figures and Tables

**Table 1:** Gemological properties of Nigerian blue-green beryl samples.

	A-B-1	A-B-2	A-B-4	A-B-6	A-B-8	C-1	C-2	C-3	C-4	C-5
Size (mm)	5.6 × 4.9 ×6.5	6.9 × 6.2 ×4.3	10.9 × 5.4 ×4.3	8.9 × 8.8 ×5.2	6.9 × 6.4 ×4.2	9.0 × 2.7 ×2.0	4.7 × 3.1 ×2.4	4.9 × 3.4 ×2.3	5.4 × 3.0 ×2.2	5.1 × 3.2 ×2.2
Weight (ct)	1.505	1.463	1.604	2.773	1.204	0.403	0.259	0.281	0.271	0.273
RI	1.568–1.574	1.571–1.579	1.573–1.580	1.571–1.579	1.577–1.583	1.567–1.574	1.572–1.578	1.574–1.582	1.569–1.577	1.567–1.574
DR	0.006	0.008	0.007	0.008	0.006	0.007	0.006	0.008	0.008	0.007
Dichroism	weak, yellowish-green/bluish-green	weak, yellowish-green/bluish-green	weak, yellowish-green/bluish-green	weak, yellowish-green/bluish-green	weak, yellowish-green/bluish-green	weak, yellowish-green/bluish-green	weak, yellowish-green/bluish-green	weak, yellowish-green/bluish-green	weak, yellowish-green/bluish-green	weak, yellowish-green/bluish-green
SG	2.6115	2.6701	2.6968	2.6791	2.7174	2.6063	2.6418	2.6952	2.6141	2.6315
UV (LW)	inert	inert	inert	inert	inert	inert	inert	inert	inert	inert
UV (SW)	inert	inert	inert	inert	inert	inert	inert	inert	inert	inert
Microscopic features	black solid inclusions, rain-like inclusions	rain-like inclusions	rain-like inclusions	black inclusions, fluid two-phase inclusions	fluid two-phase inclusions	fluid two-phase inclusions	fluid two-phase inclusions	rain-like inclusions; fluid two-phase inclusions	rain-like inclusions	black inclusions, fluid two-phase inclusions

**Table 2:** Color quantification results of the studied beryls, with color simulation via ColorTell (http://www.colortell.com, accessed 10 May 2026).

Sample Num.	L*	a*	b*	C*	h°	Color Simulation
A-B-1	34.97	−56.66	14.56	58.50	165.60	
A-B-2	58.75	−14.11	4.46	14.80	162.45	
A-B-4	55.48	−13.79	4.24	14.43	162.90	
A-B-6	49.34	−37.04	4.52	37.31	173.04	
A-B-8	95.42	−12.68	3.33	13.11	165.29	
C-1	66.74	−15.74	−2.91	16.01	190.48	
C-2	74.10	−10.40	−1.80	10.55	189.82	
C-3	85.83	−20.24	−2.12	20.35	185.98	
C-4	71.27	−14.05	−4.14	14.65	196.42	
C-5	73.82	−15.53	−5.40	16.44	199.18	

**Table 3:** Major oxide contents of the samples (wt.%).

Sample	SiO_2_	Al_2_O_3_	TFeO	Cr_2_O_3_	V_2_O_5_	MnO	CaO	K_2_O	ZnO	CuO	TiO_2_	NiO	Total (wt.%)
A-B-1	77.854	21.013	0.788	0.044	0.044	n.d.	0.032	0.111	0.005	0.011	0.021	n.d.	99.923
A-B-6	80.30	18.765	0.117	0.030	n.d.	n.d.	n.d.	0.129	n.d.	n.d.	n.d.	n.d.	99.341
A-B-4	79.742	19.363	0.205	0.045	n.d.	0.005	0.067	0.105	n.d.	n.d.	n.d.	n.d.	99.532
A-B-2	78.005	21.296	0.196	0.040	n.d.	0.005	0.041	0.081	0.026	n.d.	n.d.	n.d.	99.690
A-B-8	78.383	20.625	0.211	0.020	n.d.	n.d.	0.224	n.d.	0.036	0.009	n.d.	n.d.	99.508
C-1	77.125	21.004	1.022	0.030	0.083	0.118	n.d.	0.061	0.175	0.016	0.084	0.018	99.736
C-2	80.012	15.592	1.782	0.246	0.095	n.d.	0.531	0.129	0.018	0.01	1.149	n.d.	99.564
C-3	76.874	21.183	0.743	0.050	n.d.	n.d.	0.071	n.d.	n.d.	n.d.	0.747	n.d.	99.668
C-4	77.049	21.308	0.833	0.156	n.d.	n.d.	0.135	0.074	0.004	n.d.	n.d.	n.d.	99.559
C-5	77.801	20.521	0.59	0.097	0.122	0.165	0.427	0.218	n.d.	0.005	n.d.	n.d.	99.946

n.d., not detected under the analytical conditions used in this study; this designation is qualitative and does not indicate zero concentration. A matrix-specific quantitative detection limit for V_2_O_5_ was not established for the present semiquantitative EDXRF analysis. Because Be cannot be measured reliably by this method and natural crystal surface conditions may affect semiquantitative results, the values in Table 1 are intended for comparison of detectable components rather than complete compositional or mineral-formula calculation.

**Table 4:** Chromophoric element contents at each point of A-B-1.

	P0	P1	P2	P3	P4	P5	P6
Cr_2_O_3_	0.287	0.161	0.301	0.099	0.191	0.267	0.123
TFeO	1.251	2.149	1.900	1.868	2.048	2.126	1.631
V_2_O_5_	0.157	0.179	0.202	0.142	0.163	0.163	0.137

**Table 5:** Assignment of UV–Vis–NIR absorption bands in Nigerian blue-green beryl.

Peak Position (nm)	Assignment	References
370	Fe^3+^ d–d transition; spin-forbidden transition of octahedral Fe^3+^, accompanied by the 427 nm band	[3,32]
427	Composite absorption from Cr^3+^ and V^3+^, with superimposed Fe^3+^ d–d transition; main absorption bands of V^3+^ and Cr^3+^ overlap with Fe^3+^ transitions	[3,31,32,34,35]
611	Cr^3+^ and V^3+^ absorption; Fe^2+^–Fe^3+^ intervalence charge transfer (IVCT)	[3,34,36,37]
644/660/683	Characteristic Cr^3+^ absorption; 683 nm is the Cr^3+^ R-line, with weaker accompanying peaks at 660 and 644 nm	[3]
828	Mainly Fe^2+^ d–d transitions; overlapping broad bands from Fe^2+^ at different sites (octahedral, channel, tetrahedral)	[2,36,38,39]
955/978	Two interpretations: (1) d–d transition of channel Fe^2+^; (2) vibrational absorption of H_2_O molecules	[3,31]

**Table 6:** Chromaticity centroid parameters and color differences of the “V not detected” and “V-bearing” groups (When A-B-1 is included, the V-bearing centroid is (L* = 62.41, a* = −24.58, b* = 1.11). The direction of the a* shift relative to the “V not detected” group reverses compared with the case excluding A-B-1,undetected V was treated as 0 wt%, but this does not imply that the actual V content is zero, the ratio is expressed as oxide weight percent).

Group	n	Centroid L*	Centroid a*	Centroid b*	Hue Angle h (°)	Centroid C*	ColorTell-Simulated Centroid Color
V not detected group	6	69.35	−18.65	+1.72	174.7	18.73	
V-bearing group	3	71.55	−13.89	−3.37	193.6	14.29	
Color difference		ΔL* = +2.20	Δa* = +4.76	Δb* = −5.09	Δh ≈ +18.9°	ΔC* = −4.44	

Color differences of the “V not detected” and “V-bearing” groups: Three-dimensional color difference: ΔE*ab(3D) = √[(2.20)^2^ + (4.76)^2^ + (−5.09)^2^] ≈ 7.31; Two-dimensional color difference: Δab*(2D) = √[(4.76)^2^ + (−5.09)^2^] ≈ 6.97.

**Table 7:** Chromaticity centroid parameters and color differences of the “high-TFeO/(Cr_2_O_3_ + V_2_O_5_)” and “low-Fe/(Cr + V) groups”(undetected V was treated as 0 wt%, but this does not imply that the actual V content is zero, the ratio is expressed as oxide weight percent).

Group	n	Centroid L*	Centroid a*	Centroid b*	Hue Angle h (°)	Centroid C*	ColorTell-Simulated Centroid Color
Low-TFeO/(Cr_2_O_3_ + V_2_O_5_) group	4	59.35	−20.12	+1.96	174.4	20.21	
High-TFeO/(Cr_2_O_3_ + V_2_O_5_) group	5	78.67	−14.62	−1.53	186.0	14.70	
Color difference		ΔL* = +19.32	Δa* = +5.50	Δb* = −3.49	Δh ≈ +11.6°	ΔC* = −5.82	

Color differences of the “high-TFeO/(Cr_2_O_3_ + V_2_O_5_)” and “low-TFeO/(Cr_2_O_3_ + V_2_O_5_)” groups: Three-dimensional color difference: ΔE*ab(3D) = √[(19.32)^2^+ (5.50)^2^+ (−3.49)^2^] ≈ 20.39; Two-dimensional color difference: Δab*(2D) = √[(5.50)^2^ + (−3.49)^2^] ≈ 6.51.

## Data Availability

The original contributions presented in this study are included in the article. Further inquiries can be directed to the corresponding author.
